# Drug-Induced Type 1 Brugada Pattern: A Case Report

**DOI:** 10.7759/cureus.74282

**Published:** 2024-11-22

**Authors:** Omar Al-Anee, Maha Theeb

**Affiliations:** 1 Internal Medicine, University Hospitals Sussex NHS Foundation Trust, St Richard’s Hospital, Chichester, GBR

**Keywords:** antidepressant, arrhythmia, brugada syndrome, dosulepin, ecg (electrocardiogram), mutations, scn5a

## Abstract

Brugada syndrome is a rare genetic disorder, classified as an autosomal dominant inherited cardiac sodium channelopathy. It is associated with a high incidence of syncope and sudden death due to ventricular tachycardia or ventricular fibrillation in patients with structurally normal hearts. This report presents the case of a 33-year-old male who experienced recurrent syncopal episodes over the course of a year. The episodes resembled seizure activity, with associated memory loss of the events and no preceding warning symptoms. The patient was ultimately diagnosed with a drug-induced type 1 Brugada pattern, triggered by the antidepressant dosulepin. His medical history was significant for psychiatric conditions, including depression and previous suicide attempts, but there was no history of known cardiac conditions or epilepsy, nor any family history of sudden cardiac death. The patient denied using illicit substances or consuming significant amounts of alcohol. The physical examination was unremarkable. Investigations revealed a troponin level of less than 3.3 ng/mL, and an ECG showed cove-shaped ST-segment elevation with inverted T-waves in leads V1-V2, consistent with a type 1 Brugada pattern. Imaging, including a CT head scan, chest X-ray, echocardiogram, and blood tests (full blood count, electrolytes, and CRP), were all within normal limits. A neurological assessment suggested that the syncopal episodes were unlikely to be of neurological or epileptic origin, with a normal EEG. The cardiology team reviewed the case and agreed that the ECG findings were consistent with a type 1 Brugada pattern, with the syncopal episodes likely being caused by transient arrhythmias. Upon reviewing the patient’s medications, dosulepin, a tricyclic antidepressant, was identified as the probable trigger for the exacerbation of his underlying Brugada pattern. A psychiatry consultation was recommended to reassess this medication and explore alternative treatments. Dosulepin was discontinued and replaced with mirtazapine. Telemetry monitoring (24-hour ECG) showed normal sinus rhythm with no further Brugada pattern.

## Introduction

Brugada syndrome is a genetic condition that predisposes individuals to life-threatening arrhythmias, characterized by ECG abnormalities in leads V1-V3. Numerous genetic mutations associated with the condition have been described in the literature [1]. Brugada syndrome is classified into three types, with type 1 accounting for approximately 11% to 28% of cases and being associated with mutations in the SCN5A gene [2]. The characteristic ECG findings include a cove-shaped ST-segment elevation in leads V1-V3 of more than 2 mm in at least two precordial leads, accompanied by a negative T-wave [3]. In these patients, medications and electrolyte abnormalities can alter cardiac conduction, increasing the risk of arrhythmias. Certain medications, including tricyclic antidepressants, fluoxetine, lithium, and some antihistamines, have been linked to an increased risk of triggering type 1 Brugada syndrome, as outlined in a literature review [4]. Here, we present the case of a patient who developed recurrent syncopal episodes due to a type 1 Brugada pattern triggered by an antidepressant.

## Case presentation

**Figure 1:**
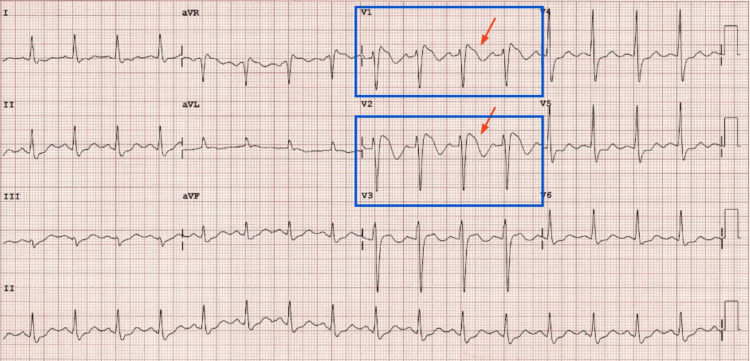
A 12-lead ECG obtained in the emergency department showing cove-shaped ST-segment elevations of more than 2 mm in leads V1 and V2, followed by a negative T wave, consistent with the type 1 Brugada pattern (indicated by red arrows)

**Figure 2:**
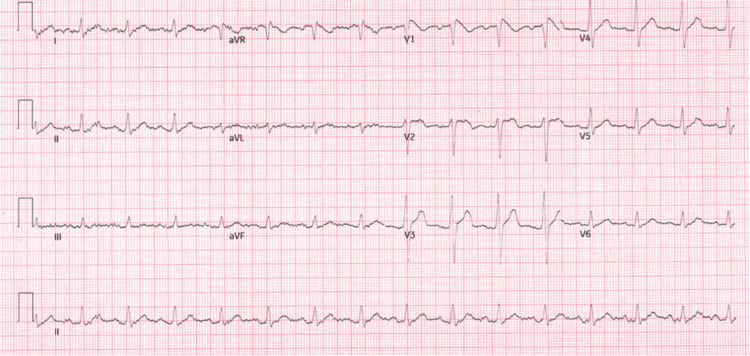
Resolution of the Brugada pattern and return to normal sinus rhythm within two days after discontinuation of the medication

A 33-year-old male presented to the emergency department with a history of four syncopal episodes over eight months. These syncopal attacks were exertional, occurring without any warning symptoms, which had not been present prior to starting the medication. Upon admission, an ECG revealed cove-shaped ST elevation in leads V1-V2, consistent with a type 1 Brugada pattern (Figure 1), followed by resolution of the Brugada pattern (Figure 2). The calculated QT interval (QTc) was 440 ms prior to stopping the medication and 400 ms afterward, showing a normal QT interval before and after discontinuing the medication. Further history revealed that the patient had recently been started on dosulepin, an antidepressant recognized by the National Institute for Health and Care Excellence (NICE) guidelines for its potential to induce cardiac conduction defects and arrhythmias. Following assessments by neurology, psychiatry, and cardiology teams, the cause of the patient’s presentation was attributed to dosulepin, which likely unmasked his underlying genetic syndrome - type 1 Brugada pattern. No evidence of ventricular tachycardia (VT) or ventricular fibrillation was found. The cardiology team decided to implant a Reveal device (implantable loop recorder) for continuous monitoring to detect abnormally fast or slow heart rhythms.

## Discussion

Some antidepressant medications can predispose patients with Brugada syndrome to develop arrhythmias, highlighting the importance of clinician awareness and the prompt cessation of triggering medications. Continuous monitoring, particularly with implantable devices like Reveal devices, is crucial for high-risk and symptomatic patients. Literature has shown that even patients with normal baseline ECGs can develop Brugada syndrome due to the side effects of medications, such as certain antiarrhythmic drugs that block sodium channels [5-7]. A multidisciplinary approach involving cardiology, neurology, and mental health teams can help improve outcomes and quality of life for patients with Brugada syndrome [8].

A study conducted in Ankara, Turkey reported that patients with a mean age of 34.9 years, predominantly males, initially presented with type 3 ECG patterns. However, following sodium channel blocker testing, most patients were diagnosed with the type 1 Brugada ECG pattern [9]. Symptoms varied with ECG type, with syncope being most common in type 1 Brugada patients.

In a study of 400 patients with Brugada syndrome, a scoring model was developed to predict arrhythmic event risk. Factors such as aborted sudden cardiac death, syncope, male gender, family history of sudden cardiac death, and specific ECG patterns were linked to a higher risk. The scoring model demonstrated strong predictive ability, with a score over 2 indicating a 9.2% chance of experiencing an event within five years [10].

Current guidelines recommend implantable cardioverter-defibrillator (ICD) placement for patients with a spontaneous type 1 ECG pattern and a history of aborted cardiac arrest or sustained VT (class I recommendation). Additionally, ICD implantation is considered for patients with syncope of arrhythmic origin due to their high risk of recurrent arrhythmias (class IIa recommendation) [11].

Professor Brugada’s study on management and diagnostic criteria emphasizes the importance of excluding other conditions that mimic the Brugada pattern. Diagnosis should also consider family history, arrhythmia-related symptoms, and ECG changes [12]. Over 300 loss-of-function mutations, mainly missense, have been identified, affecting sodium channel gating and conductance. Other genes, such as SCN1B, SCN2B, SCN3B, and GPD1L, contribute to reduced INa and Brugada syndrome, with various clinical phenotypes associated with these mutations [2].

## Conclusions

This case underscores the importance of considering genetic predispositions like Brugada syndrome in patients presenting with unexplained syncopal episodes. Awareness of medication-induced exacerbations and the prompt cessation of these drugs are crucial in mitigating the risks associated with inherited arrhythmogenic conditions, such as QTc prolongation and torsades de pointes.
